# Characterization of Immune Cells in Oral Tissues of Non-human Primates

**DOI:** 10.3389/froh.2021.821812

**Published:** 2022-02-11

**Authors:** Jamie L. Hernandez, Jaehyung Park, Sean M. Hughes, Florian Hladik, Kim A. Woodrow

**Affiliations:** ^1^Department of Bioengineering, University of Washington, Seattle, WA, United States; ^2^Department of Obstetrics and Gynecology, University of Washington, Seattle, WA, United States; ^3^Department of Medicine, University of Washington, Seattle, WA, United States; ^4^Vaccine and Infectious Disease Division, Fred Hutchinson Cancer Research Center, Seattle, WA, United States

**Keywords:** non-human primate, oral mucosa, leukocytes, buccal, sublingual, lingual tonsil, immune cell populations, flow cytometry

## Abstract

The oral mucosa contains distinct tissue sites with immune niches capable of either immunogenic or tolerogenic responses. However, immune cell compositions within oral mucosal tissues at homeostasis have not been well-characterized in human relevant tissues. Non-human primates (NHP) are a major model for the human immune system and oral anatomy, and therefore improved understanding of NHP oral immune cell populations can provide important insights for studying disease pathologies and developing therapies. Herein, we characterize immune cell types of three sites within the oral cavity (buccal, sublingual, lingual tonsil) sampled by biopsy and cytobrush in pigtail macaques. Tonsil biopsies had more T-cells, dendritic cells (DCs), DC subtypes, and CD4+ T-cells than buccal or sublingual biopsies when normalized by tissue mass. Biopsy proved to collect more immune cells than cytobrushes, however frequencies of CD45+ subpopulations were comparable between methods. Live cells isolated from biopsied tonsils had greater CD45+ leukocyte frequencies (mean 31.6 ± SD 20.4%) than buccal (13.8 ± 4.6%) or sublingual (10.0 ± 5.1%) tissues. T-cells composed more than half of the CD45+ population in sublingual tissue (60.1 ± 9.6%) and the tonsil (54.6 ± 7.5%), but only 31.9 ± 7.2% in buccal samples. CD20+ B-cells composed a greater percentage of CD45+ leukocytes in the tonsil (12.8 ± 9.1%) than buccal (1.2 ± 1.0%) or sublingual tissues (0.8 ± 1.2%). Immune population comparisons are also made between sex and age. These results present an important step for understanding the oral immune environment, oral disease, and site-specific therapy development.

## Introduction

The oral mucosa is constantly exposed to a plethora of pathogens and a diverse commensal microbiome, all of which require immune control [1]. Due to routine exposure to foreign substances like commensal bacterial and food particulates, immune cells of the oral mucosa induce a state of tolerance with regulatory T-cells (Tregs) as the main mediator of immune homeostasis [2, 3]. However, oral bacteria also generate inflammatory responses from cells like macrophages, mast cells, and neutrophils that can cause chronic oral disease. Such inflammatory responses can degrade oral tissues and are associated with autoimmune disorders and increased risk of various cancers [2]. Local macrophages and B-cells are also implicated in oral tumor development [2]. Thus, the oral mucosa is a unique immune microenvironment that plays an important role in oral health. However, despite the importance of oral immunity, the regional immune cell composition during homeostasis and disease has not been thoroughly investigated.

Upon mucosal infection or immunization, antigen presenting cells (APCs) such as dendritic cells (DCs) or B-cells can initiate immune responses by producing cytokines in response to pathogen-associated molecular patterns, then migrating to lymphoid tissues and presenting antigen [4, 5]. Immunogenic responses within the oral mucosa can therefore induce protective immunity both for local and systemic sites [6]. Regions within the oral cavity, such as the sublingual, buccal, and tonsil regions are highly permeable and rich in blood vessels, and are therefore especially interesting for studying immune and inflammatory responses which have health impacts within the oral cavity and for wellbeing in general [7]. Indeed, sublingual immunization has been reported to induce immune responses that surpass those elicited by intramuscular or intranasal immunization [8]. Across all of these tissue sites, the oral mucosa is composed of two major layers: the epithelium and the underlying lamina propria [9]. The mucosal epithelium and submucosa vary in the type of resident DCs, with Langerhans cells (LCs) found in the epithelium and subepithelial dendritic cells (SEDCs) found in the lamina propria. Langerhans cells play a critical role in inducing tolerogenic responses, whereas SEDCs effectively stimulate CD8+ T-cells and induce immunogenic responses [9].

While immune cell phenotype and function in the mouse oral mucosa have been extensively characterized [10, 11], the mouse oral anatomy and specific immune cell phenotypes vary from humans [3, 12]. Limited information exists on the composition of immune cell subsets in the human oral cavity, and the complex network of immune cell populations still needs further elucidation [3, 6, 13]. In humans, the oral cavity is comprised of the sublingual, buccal, and Waldeyer's tonsillar ring that are highly permeable and rich in blood vessels. Mice, however, do not have tonsils [3, 12]. The oral epithelium in mice is also universally keratinized, and therefore more mechanically reinforced and impermeable, while in humans only regions which experience masticatory forces (i.e., the gingiva and hard palate) are keratinized. The mucosal epithelium across the oral cavity ranges in thickness and acts as an anatomical barrier [3]. In humans, the buccal epithelium ranges from 500 to 800 μm, but is only approximately 50 μm thick in mice. More comparably, the buccal epithelium in non-human primate (NHP) models has been measured to be approximately 460 μm [14]. The NHP model has an advantage over other animal models, especially for clinical immunology studies, as *Macaca* species and humans share 93% sequence identity [15]. These similarities in genomics also yield parity in immunogenetics and age-related changes in immune functions [16, 17]. Further, structural similarities between humans and NHPs enable study of the lingual tonsil, a lymphoid tissue that is active in oral inflammation and immunity [18]. The lingual tonsil has been understudied in comparison to palatine and nasopharyngeal tonsils, as biopsies of these tissues are more readily available through routine adenotonsillectomy procedures [18]. Knowledge of regional immune cell composition in the NHP oral cavity would enable a more accurate assessment of immune cell phenotypes and functions in human oral mucosal tissues.

While immune cells are known to exist across the oral mucosa, immune cell types and compositions in the NHP oral mucosa have not been completely characterized and compared across these oral tissue sites [3, 13, 19, 20]. Here, we characterize and compare immune cell sub-populations within buccal, sublingual, and lingual tonsil tissues of NHPs by cell frequency and density, and also compare two sampling methods, biopsies and cytobrushes. Additionally, we assessed the impact of NHP sex and age. These findings provide human-relevant insight for the immune cell populations which exist and vary across the oral cavity at homeostasis. This knowledge can inform oral pathologies, and further could be applied toward therapies like vaccines which target site-specific immune cells in oral mucosa.

## Methods

### Biopsy Punch Sample Preparation and Enzymatic Digestion

Non-human primates (pigtail macaque, *Macaca nemestrina*) oral cavity tissues were donated by the tissue distribution program of the Washington National Primate Research Center (University of Washington, Seattle, WA). Donated tissues included the sublingual, buccal, and lingual tonsil from three male NHPs (age range of 11.5–20.8 years) and four female NHPs (age range of 10.2–19.8 years). Samples were selected from NHPs without conditions or interventions with known impacts on immune cell populations. Fresh tissues were biopsied four times per site by a biopsy punch (6 mm diameter, Integra Miltex, Princeton, NJ). Layers of connective tissue and muscle were excised to digest only epithelium, lamina propria and submucosa layers. Biopsies were weighed, then fully immersed into PBS for 5 min to dissolve mucus out and extensively washed by vortexing and spinning at 1,200 rpm for 5 min (4°C).

Methods for cell collection from tissues were adapted from McKinnon et al. [21]. Briefly, R15 and enzymatic digestion media were prepared fresh before each biopsy digestion, consisting of RPMI-1640, 2 mM L-glutamine, 1% (v/v) penicillin-streptomycin, and 15% (v/v) heat inactivated fetal bovine serum (Life Technologies, Carlsbad, CA). Collagenase digestion media consisted of 1 mg/ml collagenase type II and 1 unit/ml DNase I (all from Sigma, St. Louis, MO) in a 1:1 mixture of PBS and R15. Washed tissue biopsies were minced using scissors in the collagenase digestion media (all biopsies per tissue site in 3 ml digestion media) and incubated on a rotational shaker (at 200 rpm) at 37°C for 30 min, then aspirated into and expelled from a 30-ml syringe through a blunt 16 G needle 20 times and passed through a 70 μm cell strainer into fresh R15. Undigested tissue collected in the strainer was resuspended in fresh digestion media, incubated, processed by the syringe, and strained three times. Remaining undigested tissue in digestion media were processed again with the syringe, then centrifuged at 1,200 rpm for 5 min (4°C). Cells and remaining undigested tissue pieces were resuspended in 20 ml PBS, vortexed, and passed through a 40 μm cell strainer. Collected cells were resuspended in 10 ml PBS and counted.

### Tissue Sample Preparation (Cytobrush) for Direct Cell Collection

Cells were directly collected from the mucosal tissue surface using cytobrushes (Medscand Medical, Cooper Surgical, Trumbull, CT). Six spots on the tissue surface were brushed with six cytobrushes by a single 360° rotation per cytobrush. Cells were extracted by moving each brush in and out of the tip of a 25-ml serological pipette while 20 ml of PBS was gradually expelled onto a petri-dish. Collected cells were then passed through a 100 μm cell strainer. Each cytobrush was completely washed by scraping on the side wall of the strainer using additional 5 ml of PBS. The collected cell suspension was then centrifuged at 1,200 rpm for 5 min (4°C), resuspended in new 20 ml PBS, and sieved through a 40 μm cell strainer. Collected cells were resuspended in 10 ml PBS and counted.

### Cell Staining and Flow Cytometry

Samples were diluted to 10^6^ cells/ml in PBS, stained with a Live/Dead Fixable stain kit (Life Technologies, Carlsbad, CA), washed, and resuspended in FACS buffer [PBS with 1% (v/v) heat-inactivated FBS]. In separate panels for DCs and T-cells, cells were stained with fluorescently conjugated antibodies against CD1a (clone SK9; IgG2bκ), CD3 (clone SP34-2; IgG1_λ_), CD4 (clone L200; IgG1κ), CD8 (clone RPA-T8; IgG1κ), CD11b (clone ICRF-44; IgG1κ), CD20 (clone 2H7; IgG2bκ), CD45 (clone D058-1283; IgG1κ) (BD Biosciences, San Jose, CA), and CD11c (clone S-HCL-3; IgG2bκ) (Biolegend, San Diego, CA) for 30 min at 4°C in the dark. Isotype staining was performed to use as negative controls in flow cytometry analysis. After staining, cells were washed and fixed using 2% paraformaldehyde (PFA). Absolute cell counting was performed using Sphero blank calibration particles (Spherotech, Lake Forest, IL). Cells were then examined using flow cytometry (Attune NxT Flow Cytometer, Invitrogen, Carlsbad, CA). Data were analyzed with FlowJo Software (Tree Star, Ashland, OR).

### Statistical Analysis

All statistical comparisons of cell types across the different tissue sites, between methods, or between sex/age groups were assessed by mixed-effects two-way ANOVA. Significant differences in cell frequencies between tissues were further assessed using the Geisser-Greenhouse correction and Tukey's multiple comparisons test. Cell counts per tissue mass were analyzed using a log_10_-transform, with assumed sphericity followed by Tukey's multiple comparisons test. Comparisons between methods, sex, and age utilized the Geisser-Greenhouse correction and Šidák's multiple comparisons test. All analyses were conducted using GraphPad PRISM (Version 5.04, La Jolla, CA).

## Results

### The Oral Cavity Is Rich in Immune Cell Populations, With Especially Notable T- and B-Cell Populations Measured in Lingual Tonsils

**Figure 1:**
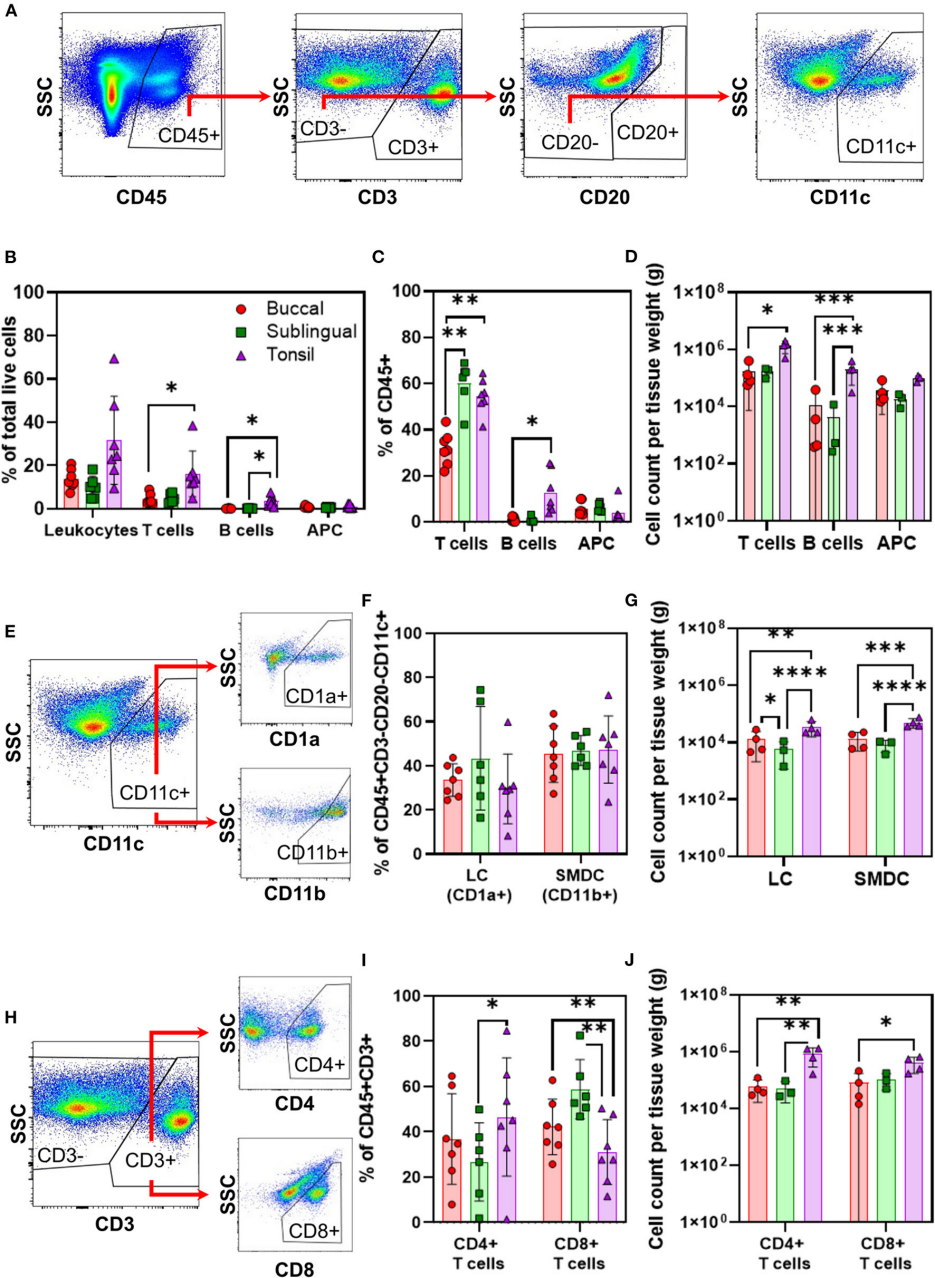
Biopsy samples from oral cavity sites show significantly different frequencies in lymphocytes but not APC populations. APCs and T-cells are present in greater frequencies per tonsil mass. Flow cytometry analysis on immune cell identification is shown for biopsy samples of buccal (red, •), sublingual (green, ■), and tonsil tissues (purple, ▲). **(A)** Representative gating strategy is shown by serial gating procedure for APCs (CD45+, CD3–, CD20–, and CD11c+ cells). **(B)** Cell frequencies (%) out of total live cells, **(C)** out of CD45+ cells, and **(D)** normalized by tissue weight are compared. LC and SEDC populations are determined by the **(E)** representative gating strategy for CD11c+ APCs, CD1a+ (LCs), and CD11b+ (SEDCs) cells, quantified by **(F)** frequencies out of CD11c+ cells and **(G)** normalized by tissue weight. T-cell subpopulations are determined by the **(H)** representative gating strategy for CD3+, CD4+, and CD8+ T-cells, quantified by **(I)** frequencies out of CD3+ cells and **(J)** normalized by tissue weight. Frequencies are plotted linearly, and cell numbers are plotted on a log scale. All data is collected by tissue biopsy, and plotted individually, and as the average value ± standard deviation, *p ≤ 0.05, **p ≤ 0.01, ***p ≤ 0.001, and ****p ≤ 0.0001. *n* = 6–7 NHPs (3 males + 4 females) for percent cell frequencies, and *n* = 2–4 NHPs for data normalized by tissue weight.

We measured immune cell frequencies in buccal, sublingual, and lingual tonsil biopsies. Immune cells were identified based on their cell surface markers and measured for viability, frequency, and counts normalized by tissue mass. Serial gating for specific leukocyte populations was performed based on CD45+ cells (total leukocytes) gated from total live cells extracted from tissue biopsies of each site (Figure 1A). The oral cavity is rich in CD45+ immune cells: all oral cavity sites had measurable levels of viable CD45+CD3+ T-cells, CD45+CD3–CD20+ B-cells, and CD45+CD3–CD20–CD11c+ APCs or non-B cell APCs (Figure 1B). T-cells were the most abundant (Table 1; Figure 1B), which is consistent with reports of human buccal and gingiva tissues [2, 13].

**Table 1:** Immune cell counts per tissue mass or brushing from biopsied and brushed oral tissue samples.

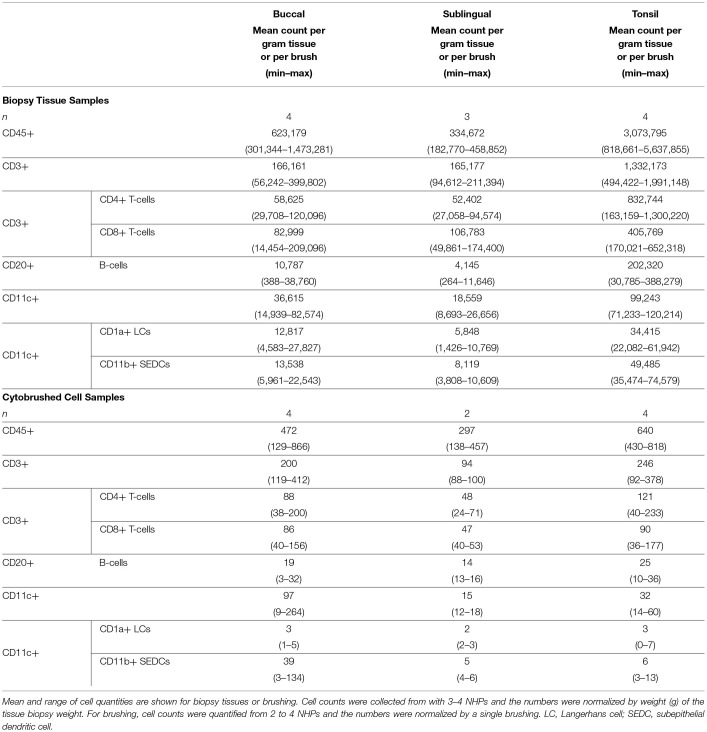

Of live leukocytes, tonsil tissues had significantly more T-cells (*p* = 0.0422) and B-cells (*p* = 0.0244) than buccal tissues (Figure 1B). Of CD45+ leukocytes, sublingual and tonsillar T-cells showed a similar frequency (60.1 ± 9.6 and 54.6 ± 7.5%, respectively) that was up to two-fold higher than in the buccal cavity (31.9 ± 7.2%, *p* = 0.0058 and 0.0053, respectively) (Figure 1C). In the buccal and sublingual sites, we measured a similar ratio of B-cell to APC frequencies (1:6). In contrast, the tonsils showed a greater frequency of B-cells to APCs (3:1). Trends of cell counts normalized by tissue mass mostly followed their population frequencies, with T-cells having the greatest density across all sites. Further, tonsil tissues also showed significantly greater T-cell (*p* = 0.0281) and B-cell (*p* = 0.0002) densities than buccal tissues (Figure 1D). Absolute immune cell quantity in tonsil biopsies exhibited the largest mean values for all immune cell types (Table 1), specifically having at least 12 times higher total T-cells compared to other tissue sites.

CD8+ T-cell frequency is highest in sublingual tissues, while CD4+ T-cells and SEDCs have the greatest density in tonsillar tissues.

Across buccal, sublingual, and tonsil tissues we further investigated subpopulations of non-B-cell APCs (CD45+CD3–CD20–CD11c+CD1a+ LCs, CD45+CD3–CD20–CD11c+CD11b+ SEDCs, representative gating shown in Figure 1E) and T-cells (CD45+CD3+CD4+, CD45+CD3+CD8+, representative gating shown in Figure 1H). We found that all tissue sites exhibited similar frequencies of LCs and SEDCs (Figure 1F). Frequencies for CD4+ T-cells were greater in tonsil than sublingual tissues (*p* = 0.0132), and correspondingly CD8+ T-cell frequencies were greater in sublingual (*p* = 0.0049) and buccal tissue (*p* = 0.0041) than tonsil tissue (Figure 1I).

While frequency provides insight to the balance of immune cells at each site, normalized counts illustrate the overall cell density in these tissues. When comparing cell counts normalized by tissue weight, tonsil tissues showed significantly greater quantities of LCs, SEDCs, and CD4+ T-cells than sublingual and buccal tissues (Figures 1G,J; Table 1). Specifically, tonsil samples had approximately six times more LCs (*p* < 0.0001), six times more SEDCs (*p* < 0.0001), 16 times greater CD4+ T-cells (*p* = 0.0032), and four times greater CD8+ T-cells (*p* = 0.0553) than sublingual tissues. Tonsil samples also had greater normalized quantities of LCs (~3x, *p* = 0.0013), SEDCs (~4x, *p* = 0.0002), CD4+ T-cells (~14x, *p* = 0.0016), and CD8+ T-cells (~5x, *p* = 0.0101) in comparison to buccal tissues.

### Biopsies Give More Cells Than Cytobrushes

**Figure 2:**
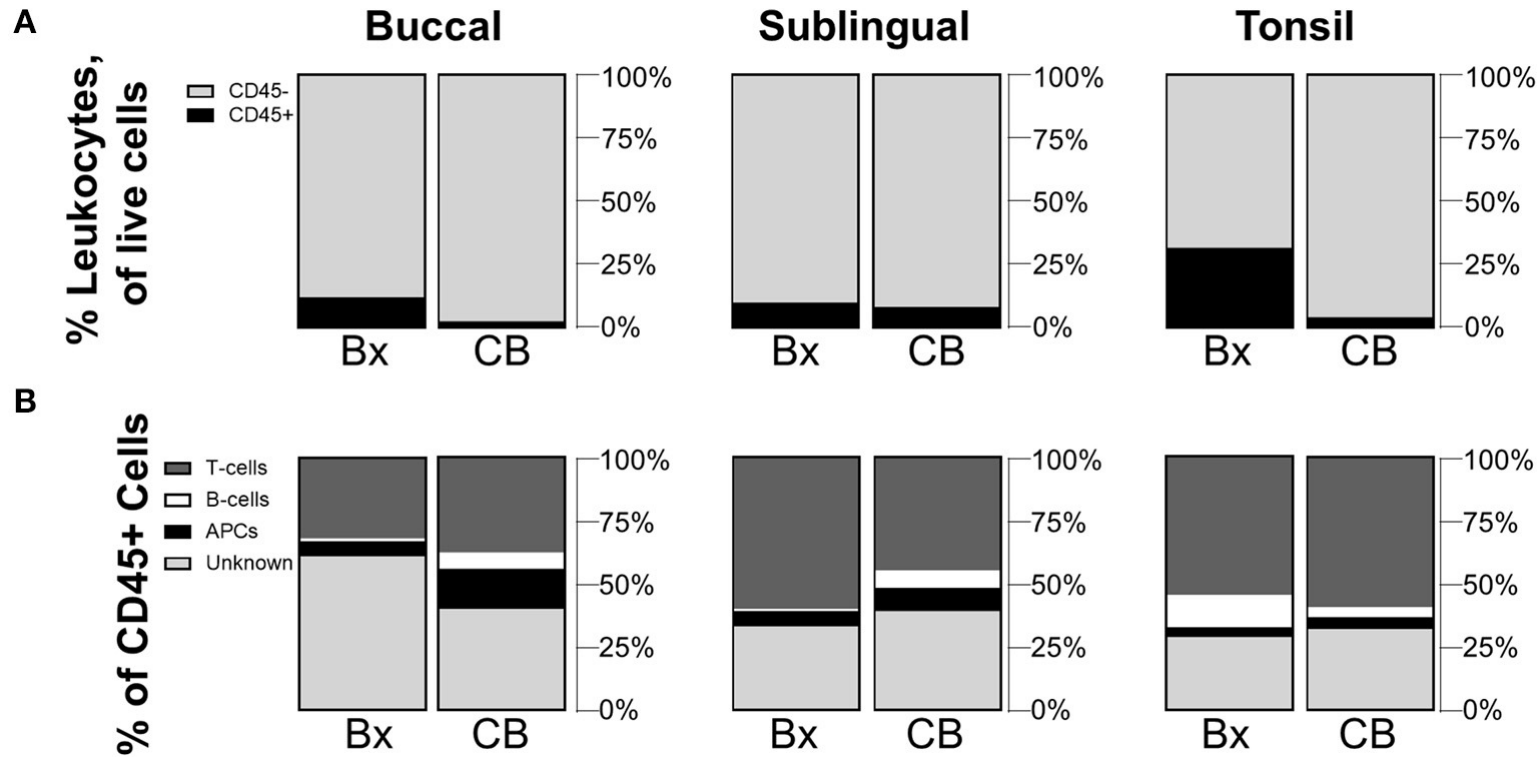
Cytobrushes yield lower frequencies of leukocytes than buccal and tonsil biopsies but show comparable distributions of CD45+ subpopulations. Compositions of **(A)** CD45+/– cells in total live cells and **(B)** subpopulations of CD45+ cells are shown by comparison between biopsy (Bx) and cytobrush (CB). Mean values of cell frequencies are used to show compositions, with *n* = 4–7 NHPs. T-cells are defined to be CD45+CD3+, B-cells as CD45+CD3–CD20+, APCs as CD45+CD3–CD20–CD11c+, and unknown immune cells are CD45+CD3–CD20–CD11c–.

Although biopsy is an effective sampling method, cytobrush sampling is less invasive and could provide a more clinically feasible method for measuring human samples, or for studying the change in cell populations in a subject over time. Since cytobrushing has shown to be an effective sampling method for other tissue sites [21, 22], we sought to compare the oral immune populations collected via cytobrush and biopsy. For all sites, cell quantities collected by the cytobrush were very small (averaging only a few hundred CD45+ cells per brushing) presumably due to limited tissue depth for brushing depending on tissue sites (Table 1). Significant differences in mean frequency values were observed between biopsied and cytobrushed samples for CD45+ cells at the buccal (*p* = 0.0274) and tonsil sites (*p* = 0.0335) (Figure 2A). When comparing CD45+ subtypes, the trends between methods are statistically similar for T-cells and B-cells (all *p*-values >0.05). However, buccal tissues showed greater proportions of APCs collected via cytobrush (*p* = 0.0412) and greater unknown leukocytes collected by biopsy (*p* = 0.0461, Figure 2B). The average quantity of unknown immune cells were 409,616 cells/g in buccal biopsies and only 156 cells/brush in cytobrushed samples (Table 1). Therefore, error due to low cell yield by cytobrush should be considered when making these comparisons.

### Biopsied Tonsil Tissues Are Statistically Similar Across Sex and Age Differences

**Figure 3:**
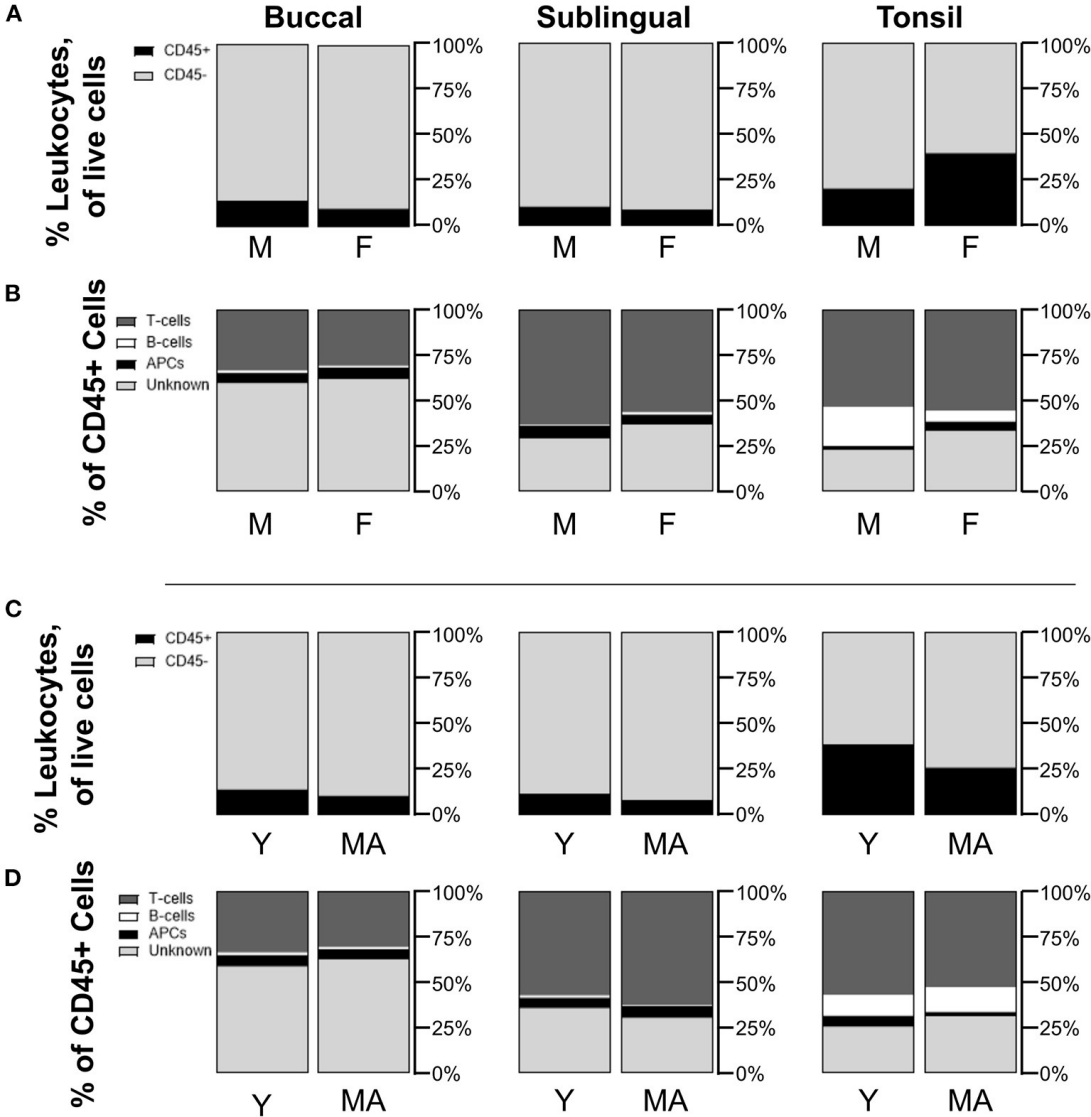
Non-statistically significant differences are measured in immune cell populations across differences in NHP sex and age. Compositions of CD45+/– cells in total live cells are shown by comparison between **(A)** male (M) and female (F) NHPs (biopsy only), and **(C)** young (Y, under 15 years) and mid-aged (MA, between 15 and 20 years) NHPs (biopsy only). Distributions of CD45+ subpopulations for **(B)** M/F NHPs and **(D)** Y/MA NHPs are defined as T-cells being CD45+CD3+, B-cells as CD45+CD3–CD20+, APCs as CD45+CD3–CD20–CD11c+, and unknown immune cells as CD45+CD3–CD20–CD11c–. Mean values of cell frequencies are used to show compositions with *n* = 3–4 NHPs (3 M and 4 F, 3 Y and 4 MA).

To gauge whether sex and age could potentially influence immune cell populations, we also compared mean cell frequencies between male and female NHPs, as well as between young and mid-aged NHPs. When comparing biopsied tissue data for all tissue sites between sexes or ages, frequency mean values were not significantly different across the sexes or ages. Although non-significant, average CD45+ frequencies in tonsil biopsies were greater in female (F 39.9 ± 23.5%/M 20.6 ± 10.1%, *p* = 0.5089) and young tissues (Y 38.9 ± 27.0%/MA 26.1 ± 15.9%, *p* = 0.8871) (Figures 3A,C). Additionally, male samples showed non-significant but notably greater B-cell populations within tonsil biopsies (F 6.3 ± 2.0%/M 21.7 ± 5.9%, *p* = 0.1072). Conversely, the greater average proportion of CD45+ cells in females is largely, but not significantly, composed of unknown immune cells (F 33.0 ± 13.2%/M 22.7 ± 3.5%, *p* = 0.5199) (Figure 3B). No notable differences in CD45+ sub-type populations are identified between the two age groups (Figure 3D). Lack of statistical significance may be an artifact of low sample size when the samples are divided by these factors.

## Discussion

Here, we identify and quantify immune cell populations across the buccal, sublingual, and lingual tonsil regions of the oral mucosa sampled from the human relevant NHP model. Biopsy led to samples with greater quantities of cells and larger populations of leukocytes, especially in tonsil and buccal tissues. The especially greater quantity of SEDCs may indicate that the tonsil is most appropriate for generating immunogenic responses. While frequency provides insight to the balance of immune cells at each site, normalized counts illustrate the overall cell density in these tissues. Our data here therefore presents two valuable quantifications of the immune microenvironment within these oral tissues. Due to the higher cell yield, we used biopsied rather than cytobrush samples as the main method for tissue comparisons. In a study of endocervical cytobrushes and biopsied ectocervical tissue, cytobrushed samples yielded similar CD45+ cell counts as the biopsy method, but the methods varied significantly in immune cell distribution. Cytobrushed samples collected more macrophages, while biopsy samples collected more T-cells [21]. While we did not stain for or quantify macrophages, we detect T-cells as the dominant immune cell population across all oral sites for both methods. Our lower cell yield from cytobrushes likely arises from the location of tissue sampling, and specifically the differences in mucosal epitheliums. While cytobrushes can collect high cell quantities from the simple columnar epithelium-lined endocervical canal [21], tissues in the oral cavity have a stratified squamous epithelium, ranging from 100 to 800 μm thick [3], which makes for a protective barrier against cell collection. Treatments to disrupt the epithelium prior to brushing could improve cell yield.

Analyses of sex and age delineated samples showed no statistically significant differences between populations. Larger immune cell populations in female and young samples were hypothesized due to known immune system sex and age differences. Greater CD4+ T-cell populations are observed in women as a result of sex steroid hormones [23], while naïve B- and T-cell production is known to decrease with age [24]. Although non-significant, female tonsil biopsy samples showed greater leukocyte and specifically unknown leukocyte populations. Such unknown leukocytes could be cells like macrophages, neutrophils, natural killer (NK) cells, or CD11b+CD11c– cells. Neutrophils and NK cells have been found to be abundant in mucosal tissues, and including macaque buccal and tonsil samples [17, 25, 26]. While greater cytotoxic NK activity has been described in human females, a difference in quantity based on sex is not characterized in the literature [27]. In mice, oral CD11b+CD11c– cells have been described as macrophage-like and inductors of immune tolerance in mice [28]. In humans, CD11b+CD11c– cells have further been described as anti-inflammatory M2 macrophages, and interestingly these CD11b+CD11c– populations have been found to be elevated within biopsy tissues and blood of patients with oral squamous cell carcinoma [29].

In general, these data support that the immune cell composition is similar between the different oral cavity sites except for a significantly greater frequency of B-cells in the tonsils compared to the other two tissue sites, and of T-cells in both tonsils and sublingual tissues compared to buccal tissues. Overall, this suggests the tonsil may be a more active tissue location for executing immunogenic responses than buccal tissue. However, Tregs and B-cells have also been implicated in the induction of tolerogenic responses [30], therefore subpopulations of APC and T-cells should be considered. Frequency measures show similar ratios of APC subpopulations in tissues, greater CD4+ T-cells in tonsil tissues, and greater CD8+ T-cells in sublingual tissues. Interestingly, this sublingual T-cell population differs from nearby gingival tissue, which is reported to have CD4+ T-cells as the dominant immune cell population [2]. However, when accounting for the overall measured quantity normalized by the mass of tissue, tonsil tissues show the greatest quantity of all APC and T-cell subtypes. This greater immune cell density further supports that lymphatic tonsil tissues may be the oral tissue site with the greatest potential to elicit immunogenic responses.

Frequency trends of CD45+ cells, and specifically T- and B-cell compositions in buccal tissue are comparable to previously reported human buccal immune cell populations. Dutzan et al. found approximately 10% of all human buccal tissue cells to be CD45+, similar to our CD45+ NHP buccal population reported here at 13.8% (Figure 1B) [13]. Of these CD45+ cells, human buccal samples were found to be approximately 45% T-cells and nearly zero B-cell counts. We similarly found NHP buccal populations to be 31.9% T-cell and 1.1% B-cell (Figure 1C). However, we report a 6% APC population within the buccal CD45+ compartment, while they report a population of DCs and macrophages closer to 30% in buccal tissues and approximately 10% in gingiva tissues [13]. Discrepancies between these results could be due to the differences in gating strategies for APCs. Here, we identified APCs as live, CD45+CD3–CD20–CD11c+ cells (Figure 1A). Dutzan et al. identified DC and macrophage populations (APCs) through gating populations of cells with high major histocompatibility complex class II expression (HLA-DR) and mid to high cell-granularity as measured by side scatter (SSC) [13]. In human tonsil tissues collected from routine tonsillectomies, Leelatian et al. detected approximately 35.8% T-cells of CD45+ cells and 95% HLA-DR+, APC population within non-T-cells. While T-cell populations are relatively comparable, this larger (~4 vs. 59% of CD45+ cells) APC population likely includes additional cells like B-cells. Further, these tissues are likely palatine tonsils, while our analysis is conducted with lingual tonsil samples [31]. To the best of our knowledge, comparable studies do not exist for sublingual tissues.

Our study can provide fundamental information needed in the development of novel oral mucosal immunotherapies. Jones et al. found vaccine delivered simultaneously to buccal and sublingual tissues elicited strong IgG responses in serum and in mucosal secretions [32]. Unfortunately, tolerogenic, rather than immunogenic, responses to these vaccine antigens are also frequently observed. Sun et al. reported tolerogenic responses following sublingual vaccination, generated by B-cells which promoted Treg expansion [30]. Langerhans cells have been identified as the main target for oral mucosal vaccination, yet various studies have also implicated epidermal LCs in the induction of antigen tolerance [3, 9, 10]. Subepithelial dendritic cells conversely have been associated with greater immunogenicity [3]. Therefore, cell composition of the specific tissue region must be considered with the intention of inducing immunogenic or tolerogenic responses. Considering the significant density of SEDCs in tonsil tissues, this tissue site may be expected to best induce immunogenic responses. However, a large density of LCs is also detected, which may lead to tolerance if antigen uptake occurs by cells at the mucosal surface. Therefore, in investigations of oral therapies like vaccines, administration route and the specified location should be considered. In addition to targeting appropriate immune cell populations, bypassing the protective layers of epithelium is needed for effective agent uptake by tissues and specifically SEDCs for induced immunogenicity. Indeed, cytobrush sampling in our study shows limited access to immune cells at the mucosal surface. Future applications of this research may motivate the use of tissue-specific therapies which can bypass the epithelium, such as microneedle systems.

The use of NHP samples here can also support future study of oral disease pathologies and development of novel therapeutics, as macaque models are immunologically similar to humans. Specifically, surface markers on NHP and human immune cells are known to be structurally similar wherein anti-human antibodies can successfully recognize the equivalent cell surface receptors in macaques. For example, human and macaque DCs express identical toll-like receptors, which are different for mice [15]. Further, the use of NHP models in studies of mucosal immunity is beneficial, as mucosal tissues of both humans and macaques express T-cell homing receptors and vascular addressins [33]. Humans and NHPs also have common pathogens such as tuberculosis, measles, and Ebola [15]. However, this is not the case for all diseases, and NPHs like macaques have been described to have differences in susceptibility and sensitivity in responses to viral and bacterial stimuli as a result of the regulation of interferon responses [15, 34]. Non-human primate models using the closely related simian immunodeficiency virus (SIV) have proven to be a vital tool for elucidating human immunodeficiency virus (HIV) pathogenesis and investigational immunizations [15, 33]. Various immune cell similarities between these species have been discovered through these studies, including the response of specific T-cell subsets toward SIV in macaques and HIV-1 in humans [15, 33]. Insight from NHP tissue models already have proven utility for controlling human diseases, and further understanding of oral immune cell populations in these model tissues can promote the study of oral diseases and therapeutics.

## Data Availability Statement

The datasets presented in this study can be found in online repositories. The names of the repository/repositories and accession number(s) can be found below: http://dx.doi.org/10.17632/x83f4kkr9v.1.

## Ethics Statement

The animal study was reviewed and approved by University of Washington Institutional Animal Care and Use Committee (IACUC).

## Author Contributions

JH conducted experiments, contributed to data analysis, wrote, and edited the manuscript. JP designed and conducted experiments, performed data analysis, wrote, and edited the manuscript. SH and FH provided guidance with project planning and experimental design. KW provided project planning, guidance with experimental design, and wrote and edited the manuscript. All authors reviewed and provided manuscript feedback.

## Funding

Research is supported by NIH/NIDCR grant R56DE028539 to KW, the Howard Hughes Medical Institute (HHMI) (Gilliam Fellowship for Advanced Study to JH and KW), and ARCS (Achievement Rewards for College Scientists) Foundation (to JH).

## Conflict of Interest

The authors declare that the research was conducted in the absence of any commercial or financial relationships that could be construed as a potential conflict of interest.

## Publisher's Note

All claims expressed in this article are solely those of the authors and do not necessarily represent those of their affiliated organizations, or those of the publisher, the editors and the reviewers. Any product that may be evaluated in this article, or claim that may be made by its manufacturer, is not guaranteed or endorsed by the publisher.
